# Severity index for rheumatoid arthritis and its association with health care costs and biologic therapy use in Turkey

**DOI:** 10.1186/2191-1991-3-5

**Published:** 2013-03-12

**Authors:** Onur Baser, Erdem Baser, Akif Altinbas, Abdulkadir Burkan

**Affiliations:** 1 Internal Medicine Department, Rheumatology Division, The University of Michigan, 211 N 4th Avenue, Suite 2B, Ann Arbor, MI 48104, USA; 2 STATinMED Research, 211 North 4th Avenue, Suite 2B, Ann Arbor, MI 48104, USA; 3 STATinMED Research, Trump Towers, Büyükdere Caddesi, Şehit Ahmet Sokak, No: 1/405, 34010, Şişli İstanbul, Turkey; 4 Gastroenterology Clinic, Dıskapı Yıldırım Beyazıt Teaching and Research Hospital, Irfan Bastug Cad, Diskapi 06100 Ankara, Turkey; 5 Program Development, Social Security Institution, Ziyabey Cad. No 6,06520, Balgat Ankara, Turkey

**Keywords:** Rheumatoid arthritis, Severity index, Medical costs, Real-world data analysis, Outcomes research

## Abstract

**Objective:**

This study aimed to apply the previously validated severity index for rheumatoid arthritis (SIFRA) to prevalent rheumatoid arthritis (RA) groups in Turkey and determine the effect of RA severity on health care costs and biologic use.

**Methods:**

This retrospective study used the Turkish national health insurance database MEDULA (June 1, 2009-December 31, 2011). Prevalent RA patients were required to be age 18 to 99, have two RA diagnoses at least 60 days apart and be continuously enrolled 1 year prior to (baseline period) and post (follow-up period) index date, which was the first RA claim during the identification period (June 1, 2010-December 31, 2010). SIFRA was calculated for the baseline period. Total health care costs and biologic use were examined for the follow-up period. The chi-square test was used to determine the association between SIFRA score terciles and outcomes. Generalized linear models were applied to determine health care costs while multivariate logistic regression determined the effect of SIFRA on outcome measures for biologic use.

**Results:**

A total of 1,920 patients were identified. The mean SIFRA score was 14.21, and 7.05 (49.57%) of the mean composed of clinical and functional status variables, followed by 6.32 (44.47%) for medications, 0.48 (3.40%) for radiology and laboratory findings, and 0.32 (2.25%) for extra-articular manifestation. There was a significant variation in scores across cities. After controlling for age, gender, region, and comorbidity index, patients in the high SIFRA tercile were 5.16 times more likely to be prescribed biologics (p<0.001, confidence interval [CI]: 3.46-7.69), and incurred annual health care costs that were €2,091 higher (p<0.001, CI: €1,557 - €2,625) than those in the low SIFRA score tercile.

**Conclusion:**

RA severity varies throughout Turkey and is a significant determinant of health care costs and biologic therapy use. Therefore, future comparative effectiveness studies should include the severity measure in their analysis.

## Background

Rheumatoid arthritis (RA), a progressive and disabling autoimmune disease, has significant economic implications for individual patients as well as a society as a whole [1,2].

The worldwide prevalence of RA has been estimated at 1%, but tends to be higher in elderly populations [3]. RA prevalence in the United States has been estimated at 2% for persons over the age of 60 [4,5]. There are approximately 3 million RA patients in Europe [6].

There is no curative treatment for RA and joint damage is progressive. Treatment of the diseased joints aims to slow the progression of joint damage and restore pain-free function. Prior to the advent of biologic therapies, commonly used pharmacological treatments included non-steroidal anti-inflammatory drugs (NSAIDs), and disease modifying anti-rheumatic drugs (DMARDs), such as methotrexate, injectable gold salts, sulfasalazine and leflunomide [7]. The introduction of biologic treatment has transformed the expectations of RA management. These medications have proven effective in slowing disease progression, achieving sustained remission, and minimizing disease activity [8]. Although effective, biologics are an extremely expensive form of therapy [9]. The use of biologic agents among newly diagnosed RA patients has increased markedly over time, rising from 3% in 1999 to 26% in 2006 [10]. Therefore, in the last decade, health care costs and utilization of patients with RA have been increasingly recognized.

Costs associated with RA have been estimated at $8.7 billion annually in the United States [11]. RA is responsible for 250,000 hospitalizations and more than 9 million physician visits per year [12,13]. Since RA patients have a peak onset near age 40 and often live for more than 30 years with joint issues that adversely affect their function, there are significant indirect costs associated with RA [14]. In Europe, 32% of the average annual RA costs were associated with indirect costs [2]. In a recent study, 71% of the overall RA-related costs in Turkey were associated with indirect costs [15].

An increasing proportion of the aging population [16], combined with effective but expensive RA treatment options, is resulting in the need for disease-specific techniques to estimate costs. Although many observational studies have attempted to estimate the burden of RA, a key limitation is the lack of disease severity measures in datasets [17-20].

This study used a previously validated claims-based severity index for rheumatoid arthritis to estimate the distribution of disease severity across regions in Turkey [21-23]. As a secondary objective, the association between disease severity and health care costs and utilization was analyzed among patients diagnosed with RA in Turkey. Finally, the study also examined the relationship between disease severity and biologics use.

## Methods

A retrospective analysis was performed using medical and pharmacy claims and eligibility data from the research identified MEDULA dataset for diagnosed RA patients in Turkey. The MEDULA dataset encompasses 17,800 pharmacies, 5,600 general practitioners, 4,500 medical centers, 1,200 government hospitals and 338 private hospitals covering more than 80% of the Turkish population.

**Figure 1:**
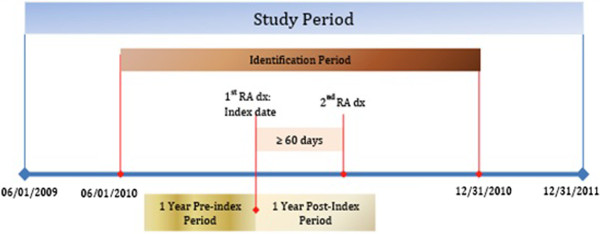
Study period.

All patients diagnosed with RA were identified using the appropriate diagnosis codes from the International Classification of Disease Tenth Revision Clinical Modification (ICD-10-CM) for the identification period (June 1, 2010 - December 31, 2010; Figure 1). Patients were required to have two RA diagnoses at least 60 days apart. The requirement of two visits and a period between claims has been shown to increase the reliability of the RA diagnosis [14]. The date of the first diagnosis claim was designated as the index date. All patients were required to be at least age 18 years on the index date and continuously enrolled in the health plan throughout the 1-year pre- (baseline) and 1-year post-index (follow-up) periods. Since this study particularly focused on prevalent patients, an RA diagnosis during the baseline period was required. All medical claims were compiled over the study period for all remaining patients.

The severity index for rheumatoid arthritis (SIFRA) score was derived for each patient. SIFRA is composed of a set of 28 RA-related indicators from a study by Cabral *et al*. [22]. These indicators were sub-grouped as clinical and functional status, extra-articular manifestations, surgical history and medications (Table 1). The strength of each relationship was measured from 0=no relationship, to 6=perfect relationship and assessed by six board-certified, clinically active rheumatologists according to the Delphi panel method. The index was validated and applied to the U.S. Department of Veteran Affairs, Veterans Health Administration (VHA) data [21,23].

**Table 1:** SIFRA indicators

	**Rating 1**
**Radiology and Laboratory Findings**	Mean (Range)
Presence of RF Ever	2.7 (2–4)
Presence of HLA Subtype	3.0 (1–4)
C1-2 Subluxation	5.2 (5–6)
Presence of CCP Ever	3.0 (2–4)
**Clinical and Functional Status Measures**	
Number of Rheumatologist Visits	3.2 (2–4)
**Extra-articular Manifestations**	
Pulmonary Nodules	3.5 (3–5)
Subcutaneous Nodules	2.8 (2–4)
Vasculitis Ever	5.0 (4–6)
Felty's Syndrome Ever	5.0 (4–6)
**Surgical History**	
Cervical Spine Fusion	5.5 (5–6)
Foot Joint Replacement	4.2 (2–6)
Total Hip Replacement	5.2 (4–6)
Total Knee Replacement	5.2 (4–6)
Elbow Replacement	5.3 (4–6)
Shoulder Replacement	5.5 (5–6)
**Medications**	
Any Oral Glucocorticoid Use	2.8 (2–4)
Intra-articular Glucocorticoids	2.7 (2–4)
**Disease-modifying Anti-rheumatic Drugs**	
Azathioprine	4.2 (3–5)
Cyclosporin	4.5 (4–5)
Hydroxychloroquine	2.0 (2)
Leflunomide	3.8 (3–4)
Methotrexate	3.8 (3–4)
Sulfasalazine	2.3 (2–3)
**Biologic Therapies**	
Adalimumab	4.2 (4–5)
Infliximab	4.0 (3–5)
Etanercept	4.2 (4–5)
TNF (ADA, ETN, IFX)	4.1
Non-TNF (ABA, RTX)	4.8

*RF*=rheumatoid factor; *HLA*=human leukocyte antigen; *C1-2*=cervical spine 1-2; *CCP*=anti-cyclic citrullinated peptide; *TNF*=tumor necrosis factor; *ADA*=adalimumab; *ETN*=etanercept; *IFX*=infliximab; *ABA*=abatacept; *RTX* = rituximab.

Note: For new biologic therapies approved since the “Delphi panel”, average scores were applied depending on class. For adalimumab, infliximab, etanercept, average of TNF blockers, a rating of 4.1 was applied; for abatacept and rituximab, average of non-TNF (4.8) was applied. Also medium and small joint fusion (4.825) and large joint fusion (5.3) was applied.

**Figure 2:**
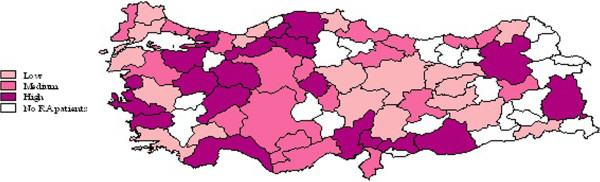
Rheumatoid arthritis severity distribution (by SIFRA terciles) across cities in Turkey.

Demographic variables such as patient gender and age were available in the data. Since Turkey is divided into seven regions, flags were created according to patient region of residence. For each city, the proportion of patients with the highest SIFRA tercile score was calculated. These proportions were ranked and presented using a Turkish map to show RA severity distribution at the city level (Figure 2).

In addition to SIFRA scores, comorbidity scores using the Elixhauser index method [24] were also calculated. The Elixhauser index is defined as the sum of a comprehensive set of 30 present comorbid conditions and has been widely used to determine patient health status.

Three sets of outcomes were defined: a) total health care costs; b) total health care utilization; and c) total medication use. Total health care costs were calculated for the follow-up period as a sum of inpatient, outpatient, pharmacy and copay amounts and adjusted to 2011 costs. Hospitalization, outpatient visits, rehabilitation visits and surgery rates were also estimated for the follow-up period. In terms of medication use, rates of biologics use were calculated for the follow-up period.

The association between SIFRA terciles and outcomes were assessed using the Chi-square test.

In order to estimate the effect of the severity score on health care costs, generalized linear models (GLMs) were used [25]. Following the Park test, the Gamma distribution with log link was selected [26]. The logistic regression model was used to determine binary outcomes. All statistical analyses were conducted using SAS v.9.3 and STATA v.11 software.

## Results

For the 1,920 RA patients identified, SIFRA scores ranged between 0 and 69.40, with a mean value of 14.21, and a standard deviation (STD) of 10.26. Mean SIFRA scores, (Table 2) were 7.05 (49.57%), which composed of clinical and functional status variables, followed by 6.32 (44.47%) for medications, 0.48 (3.40%) of radiology and laboratory findings, 0.32 (2.25%) for extra-articular manifestations (pulmonary nodules, subcutaneous nodules, vasculitis ever, and Felty’s syndrome ever), and 0.04 (0.31%) for surgical history (cervical spine fusion, hand/foot joint replacement, foot joint/ankle/wrist fusion and total hip/knee/elbow/shoulder replacement).

**Table 2:** SIFRA score distribution

**Variable**	**N**	**Mean**	**Min**	**Max**	**Median**	**STD**
SIFRA with Laboratory Data (SIFRA1)	1,920	14.21 (100%)	0.00	69.40	12.10	10.26
Radiology & Laboratory Findings	1,920	0.48 (3.40%)	0.00	6.00	0.00	1.15
Clinical & Functional Status	1,920	7.05 (49.57%)	0.00	54.40	3.20	8.50
Extra-articular Manifestations	1,920	0.32 (2.25%)	0.00	10.00	0.00	1.21
Surgical History	1,920	0.04 (0.31%)	0.00	10.03	0.00	0.50
Medication	1,920	6.32 (44.47%)	0.00	19.30	6.60	3.65

*SIFRA*=severity index for rheumatoid arthritis; *Min*=minimum; *Max*=maximum; *STD*=standard deviation.

Figure 2 shows the distribution of patients with the highest tercile of SIFRA scores across cities. The top four cities with the highest density of severe RA patients were Bolu (84%), Kocaeli (63%), Antalya (59%) and Izmir (52%).

**Figure 3:**
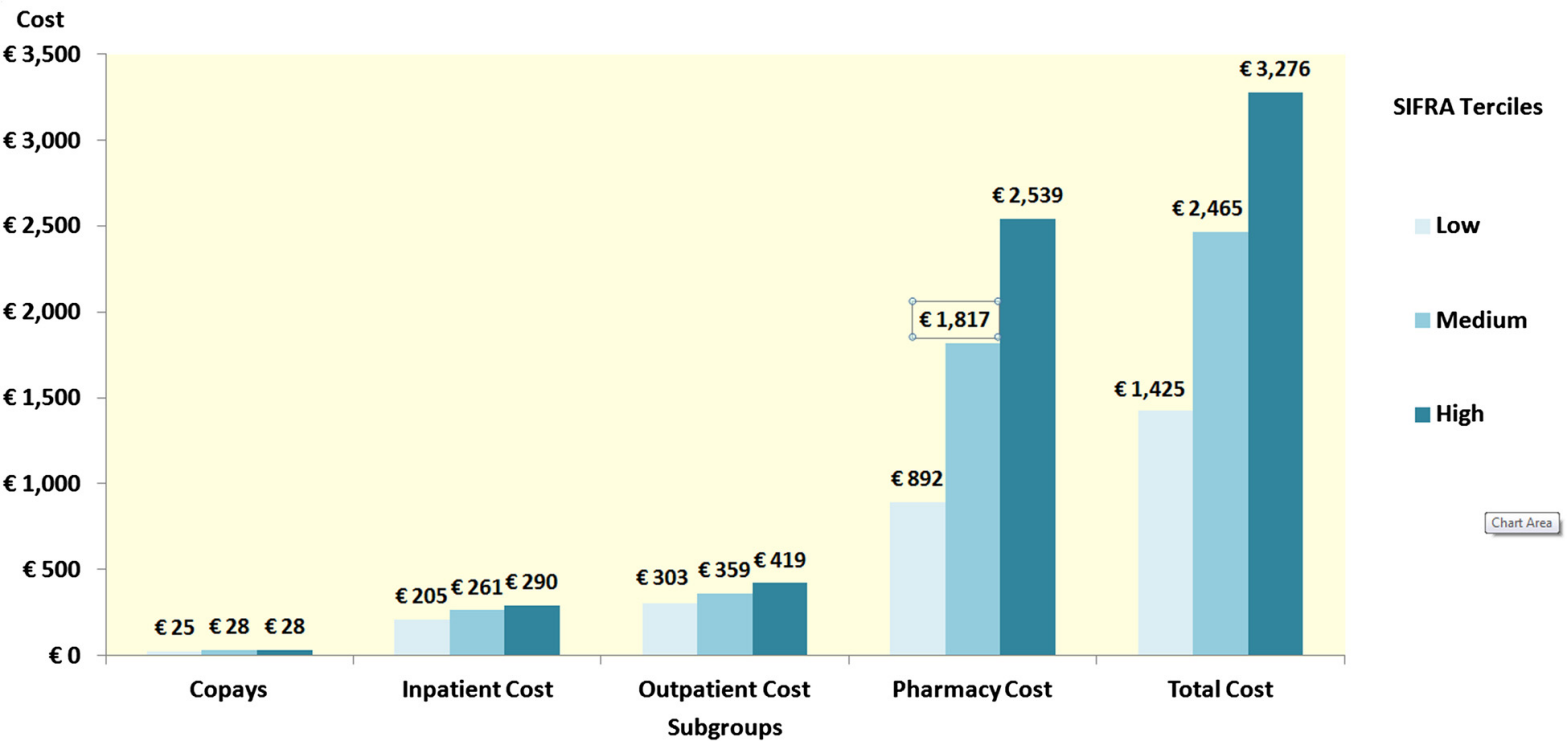
SIFRA scores and health care costs.

Total costs, as well as components of total health care costs (copays, inpatient, outpatient, pharmacy costs) by SIFRA score tercile are presented in Figure 3. Histograms indicate that patients in the upper tercile of SIFRA incurred €1,851 higher health care costs, €1,647 higher pharmacy costs, €116 higher outpatient costs, and €85 higher inpatient costs. The difference in copayments was approximately €3.

**Figure 4:**
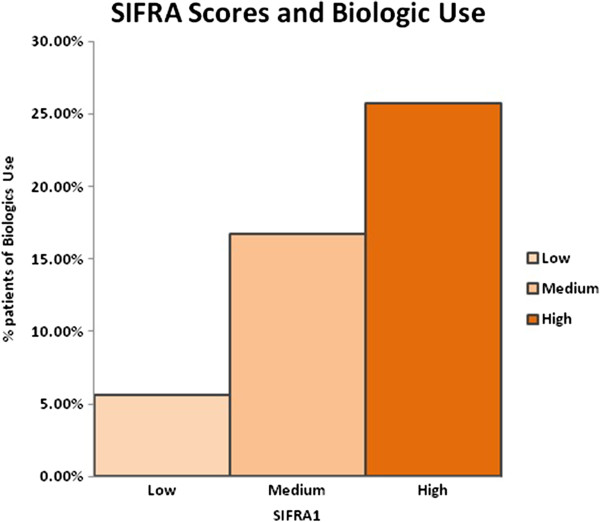
Association between SIFRA Scores and biologic use.

Figure 4 presents the relationship between baseline SIFRA scores and the use of biologic therapies in the follow-up period. The study results confirm the expectation of biologic use in more severe patients. Roughly one quarter (25.75%) of the patients in the upper tercile of SIFRA were prescribed biologics, whereas only 5.6% of patients in the lowest SIFRA tercile were prescribed biologics in the follow-up period. (p<0.001)

Using multivariate analysis, the effect of SIFRA scores on health care costs, hospitalization and biologics use was estimated. After controlling for age, gender, region, and comorbidities, patients in the high SIFRA tercile were 5.16 times more likely to be prescribed biologics (p<0.001, CI: 3.46-7.69) and incurred annual health care costs that were €2,091 greater (p<0.001, CI: €1,557- €2,625) than for those in the lower SIFRA tercile.

## Discussion

Randomized clinical trials (RCT), although described as the “gold standard” to estimate treatment effects, have limitations. Small sample size, generalizability, trial costs and time restrictions limit the applicability of RCTs [27]. Observational studies can support clinical trials by providing real-world practice patterns across geographic regions, hospitals, and patient subgroups. However, the main limitation of observational studies is the lack of control for hidden bias. Hidden bias occurs when at least one variable belonging to the estimation model is missing from the data and, therefore, is not controlled for. When disease severity is missing from the data, the term is left in the error terms in risk-adjustment models, causing bias in the estimates. Although there are advanced techniques to statistically handle hidden bias, complex models often require additional variables that are rarely available in datasets [28,29].

Due to the advancement in therapeutic options for RA patients and the high cost required to utilize them, the need for cost effective treatment methods is significant, particularly in the expanding aging population. RA expenditures are estimated at €9,946 in Belgium [18], €5,029 in the Netherlands [17], €4,000 in France, and €2,312 in Germany [16]. Overall, the estimated cost in Europe was calculated at €2,835, excluding pharmacy expenditures. Previous studies that attempted to estimate the costs of RA in Turkey were based on expert reports, local estimates and questionnaires. A recent study by Malhan *et al*. [15] estimated total annual medical costs per RA patient at €2,917. An earlier study, using data collected from hospital bills, estimated the annual cost to be €2,669. Other RA studies mostly pertained to disease prevalence and epidemiology in Turkey.

However, due to a lack of severity measures, the estimates from these prior studies were interpreted with caution. In some of the studies, severity was proxied with comorbidity index values, which were not specifically designed for RA. These indexes showed low correlation with the severity of RA.

A recent publication attempted to create a claims based, validated severity index for rheumatoid arthritis [23]. This index correlated highly with a record-based index score (RARBIS) and increased the prediction power of the models. This research applied the severity index on health care costs and utilization in patients with RA in Turkey, using nationwide, real-world data to determine the association between RA severity and health care outcomes (such as cost, utilization and biologic use) in Turkey.

Note that one of the indicators in severity index for rheumatoid arthritis (SIFRA) are biological therapies. In order to estimate the effect of RA severity on biologic use, on needs to create this index using baseline use of biological therapies. All of the indicators should be calculated in the baseline to create the index otherwise one might have endogeneity problem in estimating the models. Thus, the estimators would be biased.

Although this study controlled for severity by using the claims-based severity index for RA, there are other limitations in this analysis, which are typical of any claims-based data. Since claims data are collected for payment rather than research purposes, the presence of diagnostic codes on a medical claim does not necessarily prove existence of the actual disease. However, the probability that a patient with RA diagnostic codes also has RA was reported at 95%. Two RA diagnoses occurring at least 60 days apart were applied in order to mitigate the incorrect coding and rule-out criteria. Diagnosis codes used to define comorbidities have over 90% specificity.

## Conclusion

This paper applied the previously validated RA severity scores for diagnosed patients in Turkey. According to severity scores, the total medical costs of RA patients ranged from €1,435 to €3,275. The use of biologics was positively correlated with the severity score. Since statistically omitting a variable that belongs in population models provides biased and inconsistent estimates, any comparative effectiveness studies in RA treatment should include severity scores.

## Consent

No patient identity or medical records were disclosed for the purposes of this study except in compliance with applicable law. Since the core study proposed herein does not involve the collection, use, or transmittal of individual identifiable data, patient approval/consent to conduct this study was not required.

## Competing interests

The authors declare that they have no competing interests.

## Authors’ contributions

OB had full access to all of the data in the study and had taken responsibility for the integrity of the data and the accuracy of the analysis. Study concept and design were carried out by OB. Acquisition of data was made by AB. Analysis and interpretation of the data were carried out by OB. OB drafted the paper. OB, AB, EB, and AA made critical revisions to the manuscript for important intellectual content. Statistical programming was conducted by EB. Administrative, technical, or material support was provided by OB. OB was responsible for the study supervision. All authors read and approved the final manuscript.
